# Latent class analysis of depression and anxiety among medical students during COVID-19 epidemic

**DOI:** 10.1186/s12888-021-03459-w

**Published:** 2021-10-12

**Authors:** Zhuang Liu, Rongxun Liu, Yue Zhang, Ran Zhang, Lijuan Liang, Yang Wang, Yange Wei, Rongxin Zhu, Fei Wang

**Affiliations:** 1 School of Public health, China Medical University, Shenyang, Liaoning China; 2 Early Intervention Unit, Department of Psychiatry, The Affiliated Brain Hospital of Nanjing Medical University, 264 Guangzhou Road, Nanjing, Jiangsu 210029 People’s Republic of China; 3 School of Medical Engineering, Xinxiang Medical University, Xinxiang, Henan China; 4 Department of Psychiatry, The First Affiliated Hospital of China Medical University, Shenyang, Liaoning China; 5 Department of Psychology, The First Affiliated Hospital of Hainan Medical University, Haikou, Hainan China; 6 Psychology Institute, Inner Mongolia Normal University, Huhehaote, Inner Mongolia China; 7 Department of Psychiatry and Radiology, The First Affiliated Hospital of China Medical University, 155 Nanjing North Street, Shenyang, 110001 Liaoning People’s Republic of China; 8 Nanjing Functional Brain Imaging Institute of Nanjing Medical University, 264 Guangzhou Road, Nanjing, Jiangsu 210029 People’s Republic of China

**Keywords:** COVID-19, Latent class analysis, Medical students, Depression, Anxiety

## Abstract

**Objective:**

The novel coronavirus disease 2019 (COVID-19) is a global public health emergency that has caused worldwide concern. The mental health of medical students under the COVID-19 epidemic has attracted much attention. This study aims to identify subgroups of medical students based on depression and anxiety and explore the influencing factors during the COVID-19 epidemic in China.

**Methods:**

A total of 29,663 medical students were recruited during the epidemic of COVID-19 in China. Depression and anxiety symptoms were assessed using Patient Health Questionnaire 9 (PHQ9) and Generalized Anxiety Disorder 7 (GAD7) respectively. Latent class analysis was performed based on depression and anxiety symptoms in medical students. The latent class subtypes were compared using the chi-square test. Multinomial logistic regression was used to examine associations between identified classes and related factors.

**Results:**

In this study, three distinct subgroups were identified, namely, the poor mental health group, the mild mental health group and the low symptoms group. The number of medical students in each class is 4325, 9321 and 16,017 respectively. The multinomial logistic regression results showed that compared with the low symptoms group, the factors influencing depression and anxiety in the poor mental health group and mild mental health group were sex, educational level, drinking, individual psychiatric disorders, family psychiatric disorders, knowledge of COVID-19, fear of being infected, and participate in mental health education on COVID-19.

**Conclusions:**

Our findings suggested that latent class analysis can be used to categorize different medical students according to their depression and anxiety symptoms during the outbreak of COVID-19. The main factors influencing the poor mental health group and the mild mental health group are basic demographic characteristics, disease history, COVID-19 related factors and behavioural lifestyle. School administrative departments can carry out targeted psychological counseling according to different subgroups to promote the physical and mental health of medical students.

## Introduction

Since December 2019, a novel coronavirus pneumonia (COVID-19) outbreak has persisted in Wuhan. The World Health Organization declared that the COVID-19 outbreak constitutes a public health emergency of international concern [1]. The outbreak of the COVID-19 has caused public panic and psychological pressure [2, 3]. In January 2020, the Ministry of Education issued a notice requiring colleges to appropriately postpone school opening time. To prevent the escalation of the epidemic, schools have taken measures such as extending holidays to ensure that the majority of students are isolated in their current residences and complete their school-related responsibilities remotely [4]. For college students, extended holidays, long-term stays at home, fewer trips out of the home, and an inability to attend school and participate in social activities, may affect their academic performance and lead to their anxiety and depression [5–7].

In a recent study, the research team identified social networking as the strongest protective factor against depression and suggested that reducing sedentary activities, such as watching TV and daytime naps, could also help reduce the risk of depression [8]. This epidemic not only led to a risk of death from infection, but also led to unbearable psychological pressure. College students must reduce the frequency of their outings, resulting in their inability to participate in social activities, which may affect their learning progress and exacerbate their anxiety and depression. Therefore, the mental health status of medical students is of great concern to medical universities [9].

As a special group of future medical workers, medical students’ physical and mental development is not yet fully mature, and the healthy growth of these students can effectively promote the positive development of healthcare in the future [10]. Compared with their normal way of living and learning, staying at home was a major contrast. In fact, the epidemic has affected mental health among those in the medical industry than among those in the general public, and they must be treated correctly to adapt to this change [11]. Mental health problems may continue into adulthood if they are not detected or properly treated. For students in clinically related disciplines, these problems can lead to many undesirable personal and professional consequences [12, 13]. Therefore, it is necessary to pay attention to the mental health of medical students during the epidemic period and take targeted action to intervene with students with different characteristics.

In research on the mental health of medical students, the indirect measurement of the latent mental health can be obtained through observed and measurable behaviour. Previous studies generally used the total scores of the self-assessment scales as the standard for categorizing the mental health of medical students [14, 15]. The categorization standard was too simple to distinguish group characteristics. The application of latent class analysis (LCA) technology can solve this problem and provide more scientific methods for the classification of medical students’ mental health during epidemic. LCA is a more scientific and rigorous statistical method to classify the potential characteristics of a population based on the score probability of each item [16]. Using LCA to group depression and anxiety symptoms of medical students, considering both symptom profile and severity, is helpful to explore the potential mechanism of depression and anxiety, and to develop more targeted intervention measures.

At present, LCA has been widely used in sociology, psychology and disease classification or diagnosis [17, 18]. Current research on COVID-19 has focused on pathogenesis, epidemiology and clinical research [19–22]. There is no latent category research on mental health during the COVID-19 epidemic. Therefore, this study intends to use LCA to explore the factors influencing medical students’ mental health during the COVID-19 to provide accurate decision-making references for relevant education departments.

## Methods

### Participants

Participants in our study came from a large cross-sectional survey conducted from March to April 2020 during the COVID-19 epidemic in China. The survey selected three medical universities, and a cluster stratified random sampling method was used. Participants will be included if they are willing to participate and can complete the questionnaire on their own. Considering the severity of the COVID-19 epidemic, we collect questionnaires through the online platform rather than face-to-face interviews. Questionnaires were sent to medical students through the platform of WeChat official account in medical colleges. All the participants were informed of the purpose of this study and signed online informed consent before completing the online questionnaire. The complete questionnaires will be kept confidential and checked for mistakes and completeness through the platform. Ultimately, a total of 29,663 valid questionnaires were collected. This study was approved by the Biomedical Ethics Committee of Xinxiang Medical University (XYLL-2020235) and Hainan Medical University (HYLL-2020005), complying with the Declaration of Helsinki.

### Measures

The survey consists of five parts: basic demographic characteristics, the psychiatric history of individuals and family members, depression, anxiety, and COVID-19 related factors. Depression and anxiety are the most common mental health problems found in Chinese medical students. We focused on symptoms of depression and anxiety for all students using the Chinese versions of the following measurement tools, which have good validity and reliability.

The Patient Health Questionnaire-9 (PHQ-9) included 9 items and was adopted to screen for depressive symptoms in our study. Each item was scored from 0 to 3 (0, not at all; 1, several days; 2, more than half of all the days; 3, nearly every day), with the total scores ranging from 0 to 27. Higher scores indicated greater severity of depressive symptoms [23]. The Generalized Anxiety Disorder-7 (GAD-7) scale was a practical self-report anxiety questionnaire that comprised seven items based on seven core symptoms. The participants reported their symptoms using a 4-item rating scale ranging from 0 (not at all) to 3 (almost every day), such that the total score ranged from 0 to 21 [24]. The PHQ-9 and GAD-7 items were recoded into binary variables for the LCA.

### Statistical analysis

LCA models were conducted to identify data-driven subgroups using Version 8.2 of Mplus. The LCA can compensate for the deficiencies of factor analysis and structural equation model, which can only be analyzed with the continuous latent variables [25]. In LCA, classes are identified based on a set of categorical indicators, assuming that the latent categorical variable can explain the association among a set of observed variables [26]. In our study, we fitted one to six latent class models to determine the optimal number of latent classes.

The model fit indices used for the LCA included information criteria, the Lo–Mendell–Rubin (LMR) test, the bootstrap likelihood ratio test (BLRT), and the entropy [27]. In addition, subgroup membership interpretability is another important factor in determining the optimal model. The information criteria include the Akaike information criterion (AIC), the Bayesian information criterion (BIC) and the adjusted Bayesian information criterion (aBIC). For these fit indices, the suitable model was based on the highest entropy and the lowest AIC and BIC. The entropy is an indicator of classification accuracy, with values close to 1 indicating greater accuracy [28]. Lower AIC and BIC values indicate that the model provides a better description of the data. The LMR and BLRT are significant tests that compare model fit improvement between models with κ classes and κ-1 classes. Compared to κ-1 classes, significant *P* values suggest a better model fit with κ classes.

After the appropriate number of latent classes was identified, the medical students were assigned to their most likely subgroup based on their highest posterior class probability. Chi-square tests were conducted to examine the distribution of related factors. Multinomial logistic regression was performed to estimate the correlates of related risk factors with subtypes. Statistical significance was taken as a 2-sided *P* < 0.05.

## Results

### Demographic characteristics

A total of 29,663 medical students were investigated, including 10,185 males and 19,478 females. The average age of the medical students was 21.46 years (SD = 2.50). The demographic characteristics of medical students are shown in Table 1.

**Table 1:** Demographic characteristics of medical students

Characteristics	N (%)
**Sex**	
Male	10,185 (34.3%)
Female	19,478 (65.7%)
**Education level**	
Junior	1968 (6.6%)
Undergraduate	26,909 (90.7%)
Postgraduate	786 (2.7%)
**Smoking**	
Yes	1564 (5.3%)
No	28,099 (94.7%)
**Drinking**	
Yes	4229 (14.3%)
No	25,434 (85.7%)
**Individual psychiatric disorders**	
Yes	298 (1.1%)
No	29,365 (98.9%)
**Family Psychiatric disorders**	
Yes	340 (1.1%)
No	29,323 (98.9%)

### Model fit indices of LCA

Model fit indices for various models with different latent classes are listed in Table 2. LCA with 1 to 6 classes was performed. The results showed that the AIC, BIC and aBIC decreased with an increasing classification number. The model with 1 class had the largest AIC, BIC and aBIC, suggesting that this model fit the data the worst among the models. The 2-class model had the highest entropy value, but the LMR test was not significant. AIC, BIC and aBIC leveled off slightly after the 3-class model was assigned. Additionally, entropy values close to 1 are preferred, indicating better class separation [28]. In the 3-class and 4-class models, the LMR and BLRT values reached significance (*P* < 0.0001), but the entropy value of the 3-class model was higher, indicating that the 3-class model fit the data better than the 4-class model did. After comprehensively considering the above indicators, we selected the 3-class model because it was parsimonious and exhibited better class separation than did the solutions generated by the other classifications (Table 2).

**Table 2:** Fitness indicators of different latent class models

	AIC	BIC	aBIC	Entropy	LMR	BLRT	Class size and assignment probability
1-class	491,182.187	491,314.949	491,264.101				
2-class	365,148.853	365,422.675	365,317.802	0.929	0.3274	< 0.0001	9111 (30.72%) / 20,552 (69.28%)
3-class	341,209.321	341,624.204	341,465.305	0.888	< 0.0001	< 0.0001	4325 (14.58%) / 9321 (31.42%) / 16,017 (54.00%)
4-class	336,127.056	336,682.999	336,470.074	0.839	< 0.0001	< 0.0001	5004 (16.87%) / 2528 (8.52%) / 8642 (29.14%) / 13,489 (45.47%)
5-class	333,890.978	334,587.981	334,321.031	0.820	0.0061	< 0.0001	13,489 (45.48%) / 3263 (11.00%) / 2213 (7.46%) / 2721 (9.17%) / 7977 (26.89%)
6-class	332,306.163	333,144.226	332,823.250	0.792	< 0.0001	< 0.001	13,165 (44.38%) / 3008 (10.14%) / 6265 (21.12%) / 2767 (9.33%) / 2770 (9.34%) / 1688 (5.69%)

### Definition of latent class

The score probability of the first class was high, which showed that the medical students in this category had poor mental health status during the epidemic period and could not be effectively adjusted, so they were labeled the ‘poor mental health group’. This group had the smallest number of medical students (*N* = 4325; 14.58%). The second class (*N* = 9321; 31.42%) was defined as the ‘mild mental health group’ because it had a moderate score probability of depression and anxiety, which was lower than that observed in the first class and higher than that observed in the third class. Medical students of this type had a certain self-regulation ability during the epidemic. The third class (*N* = 16,017; 54.00%) had a low probability of scoring in all categories, indicating that this type of medical students had better mental health status during the epidemic and could effectively regulate their psychological condition, so it was named the ‘low symptoms group’.

**Fig. 1:**
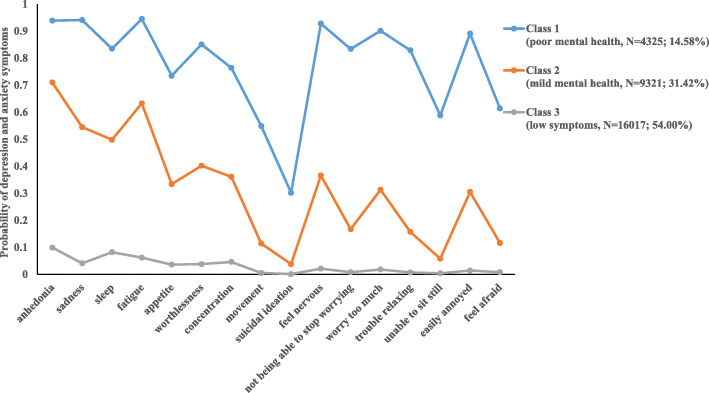
Profiles of latent classes of depression and anxiety in medical students

Figure 1 illustrates the profiles of mental health subtypes for the 3-class model. In Fig. 1, the y-axis represents the probability of depression and anxiety symptoms, while the x-axis shows indicator variables used for the LCA. The three lines showed symptom patterns for the three mental health subtypes. No crossing was observed among the three lines, suggesting that the modelled subtypes differed in symptom profiles. In particular, for the item regarding suicide or self-harm, a lower probability was shown across the three groups.

### Univariate analysis of latent class of mental health

As shown in Table 3, gender, education level, smoking, drinking, the psychiatric disorders of the individual and family members, knowledge of COVID-19, contact with confirmed or suspected patients with COVID-19, fear of being infected, and participation in mental health education on COVID-19 were significantly different across the three groups (*P* < 0.05).

**Table 3:** Univariate analysis of different latent classes of mental health status

Variables	poor mental health group (***N*** = 4325)	mild mental health group (***N*** = 9321)	low symptoms group (***N*** = 16,017)	χ2/Fisher	***P***
**Sex**				70.649	< 0.001
Male	1454	2908	5823		
Female	2871	6413	10,194		
**Education level**				76.010	< 0.001
Junior	335	499	1134		
Undergraduate	3817	8592	14,500		
Postgraduate	173	230	383		
**Smoking**				169.223	< 0.001
Yes	399	485	680		
No	3926	8836	15,337		
**Drinking**				441.697	< 0.001
Yes	990	1501	1738		
No	3335	7820	14,279		
**Individual psychiatric disorders**				53.623	< 0.001
Yes	77	118	103		
No	4248	9203	15,914		
**Family psychiatric disorders**				168.534	< 0.001
Yes	119	141	80		
No	4206	9180	15,937		
**Knowledge of COVID-19**				632.103	< 0.001
Yes	2624	6275	12,442		
No	1701	3046	3575		
**Contact suspected or confirmed**				24.006	0.001
Diagnosed	29	55	88		
Suspected	23	26	22		
No	4723	9240	15,907		
**Fear of being infected**				1701.334	< 0.001
Yes	2287	3546	3594		
No	2038	5775	12,423		
**Participate in mental health education on COVID-19**				304.553	< 0.001
Yes	2913	6861	12,714		
No	1412	2460	3303		

### Multinomial logistic regression analysis of latent classes of mental health

The category classification was used as the dependent variable, the third class (low symptoms group) was used as the reference group, and the significant factors in univariate analysis were used as independent variables for multinomial logistic regression analysis. The results showed that compared with the low symptoms group, medical students in the poor mental health group were more likely to be female (*OR* = 1.732, *P* < 0.001), have a postgraduate degree or above (*OR* = 1.740, *P* < 0.001), drink (*OR* = 2.274, *P* < 0.001), have individual psychiatric disorders (*OR* = 1.898, *P* = 0.001), have family psychiatric disorders (*OR* = 5.030, *P* < 0.001), have a lack of knowledge about COVID-19 (*OR* = 1.615, *P* < 0.001), have a fear of being infected (*OR* = 3.223, *P* < 0.001), and be unwilling to participate in mental health education on COVID-19 (*OR* = 1.397, *P* < 0.001). Meanwhile, those in the mild mental health group were more likely to be female (*OR* = 1.534, *P* < 0.001), have a postgraduate degree or above (*OR* = 1.348, *P* = 0.004), drink (*OR* = 1.869, *P* < 0.001), have psychiatric disorders of the individual (*OR* = 1.708, *P* < 0.001), have psychiatric disorders of the family (*OR* = 2.861, *P* < 0.001), have a lack of knowledge about COVID-19 (*OR* = 1.503, *P* < 0.001), have a fear of being infected (*OR* = 1.982, *P* < 0.001), and be unwilling to participate in mental health education on COVID-19 (*OR* = 1.284, *P* < 0.001) than were those in the low symptoms group (Table 4).

**Table 4:** Multinomial logistic regression analysis of different latent classes of mental health status

	poor mental health group				mild mental health group			
	*B*	*SE*	*P*	*OR* (95%*CI*)	*B*	*SE*	*P*	*OR* (95%*CI*)
**Sex**								
Female	0.549	0.050	< 0.001	1.732 (1.570–1.910)	0.428	0.032	< 0.001	1.534 (1.440–1.635)
**Education level**								
Undergraduate	0.139	0.084	0.098	1.149 (0.975–1.355)	0.334	0.058	< 0.001	1.397 (1.246–1.565)
Postgraduate	0.554	0.142	< 0.001	1.740 (1.318–2.297)	0.299	0.104	0.004	1.348 (1.100–1.652)
**Smoking**								
Yes	0.136	0.099	0.169	1.145 (0.944–1.389)	−0.072	0.072	0.321	0.931 (0.808–1.07)
**Drinking**								
Yes	0.822	0.067	< 0.001	2.274 (1.996–2.592)	0.625	0.047	< 0.001	1.869 (1.705–2.048)
**Individual psychiatric disorders**								
Yes	0.641	0.192	0.001	1.898 (1.302–2.768)	0.535	0.143	< 0.001	1.708 (1.292–2.259)
**Family psychiatric disorders**								
Yes	1.615	0.178	< 0.001	5.030 (3.552–7.124)	1.051	0.145	< 0.001	2.861 (2.153–3.804)
**Knowledge of COVID-19**								
No	0.479	0.045	< 0.001	1.615 (1.478–1.764)	0.407	0.030	< 0.001	1.503 (1.415–1.595)
**Contact suspected or confirmed**								
Suspected	0.526	0.391	0.178	1.692 (0.787–3.641)	0.564	0.309	0.068	1.758 (0.960–3.219)
Diagnosed	−0.149	0.267	0.576	0.861 (0.511–1.453)	−0.062	0.181	0.733	0.940 (0.660–1.340)
**Fear of being infected**								
Yes	1.170	0.043	< 0.001	3.223 (2.960–3.509)	0.684	0.030	< 0.001	1.982 (1.870–2.100)
**Participate in mental health education on COVID-19**								
No	0.334	0.048	< 0.001	1.397 (1.272–1.534)	0.250	0.032	< 0.001	1.284 (1.205–1.369)

## Discussion

In the current study, LCA was used to classify medical students’ depression and anxiety during the COVID-19 epidemic. LCA is an important research method in social science that assumes that individuals can be grouped into classes with similar patterns of some behaviours according to their response to a set of observed indicators [29]. Three interpretable subtypes of depression and anxiety based on LCA models were detected in the present analysis, and the entropy of the 3-class model (0.88) indicated excellent membership classification. This is consistent with previous reports involving LCA, which classified child mental health at the population level and determined the reliability of identified classes [30–32]. Meanwhile, a number of researchers have published papers encouraging the use of LCA in the classification of mental health issues because it is well suited to addressing pertinent questions [33–36]. For example, Essau CA encouraged the application of LCA for studying complex multidimensional phenomena, such as mental disorders, because multiple aspects of individual functioning can be studied holistically [37]. Other researchers have suggested that LCA is an important analytic tool for studying health risk behaviours in college students [38–42]. Furthermore, it can also be used to examine the clustering of modifiable health risk behaviours and to explore the relationship between these identified clusters and mental health outcomes [43].

This study found that the mental health of medical students had obvious grouping characteristics during the COVID-19 pandemic, and the statistical indicators supported three latent classifications, namely, the ‘low symptoms group’, the ‘mild mental health group’ and the ‘poor mental health group’. Most medical students in this study belonged to the ‘low symptoms group’, and they had low probability scores for each factor of depression and anxiety, which showed that most medical students had strong psychological adjustment ability and adaptability in isolation at home during the epidemic period. Through the probability score plot, it can be seen that all the medical students had a lower probability of scoring on suicidal ideation. It is possible that the students were in a sensitive period of youth and had more or fewer psychological problems, but they did not have ideas of self-harm or suicide.

In the poor mental health group, the probability score plot showed that the mental health problems of medical students occur in clusters rather than independently. The poor mental health group had a higher probability of scoring on all other factors except suicidal ideation, which can partly be attributed to the stressful training experience [44], such as the long length of schooling, academic pressure, and the stress of clinical practice [45]. This subtype of students may have multidimensional psychological problems, with long-term consequences on well-being and professional relationships. This is in accordance with previous studies showing that most of the students with depression symptoms were also diagnosed with generalized anxiety symptoms [46, 47]. The cause of co-existence was related to sharing the same risk factors and symptoms [48–50]. The symptoms of depression and anxiety in medical students may include slowness of thought, decreased energy, low self-worth, disturbed sleep, and difficulty concentrating, which have been known to jeopardize academic development [51, 52]. To prevent their behaviour from becoming extreme, these students urgently need corresponding psychological treatment measures and should be the focus of prevention and treatment. Computer-delivered cognitive behavior therapy (CCBT), which has become widely used for the growth of the internet and smartphones, can be considered [53, 54].

Multinomial logistic regression analysis showed that compared with the ‘low symptoms group’, there were more females in the ‘poor mental health group’ and the ‘mild mental health group’. In particular, the risk of female students entering the ‘poor mental health group’ was 1.732 times higher than that of male students, indicating that the mental health problems of female students were more prominent, which may be due to the different hormones and stressor events. Consistent with previous studies, gender differences have always existed in the mental health of medical students [55–57]. In an investigation of the effects of different educational levels, it is found that the higher one’s educational level is, the higher the risk of entering the ‘poor mental health group’ and ‘mild mental health group’. Medical students with many years of education are more likely to have psychological problems, which may be related to the higher pressure from scientific research and work [58]. Similarly, medical students with drinking habits also have a higher risk of psychological problems, which was in accordance with the findings of previous studies [59, 60]. Compared with the low symptoms group, medical students in the high-risk group with individual or family psychiatric disorders had a higher risk of mental health problems than did students without psychiatric disorders. A history of psychiatric disorders was consistently found to be significant correlate of depression and anxiety [61, 62].

Apart from traditional factors, epidemic-related factors were also observed in our study. Compared with the low symptoms group, the higher the awareness of COVID-19, the lower the risk of psychological problems for medical students in the poor mental health group and mild mental health group. This phenomenon elucidated that the better understanding of preventive measures about COVID-19 for medical students, the more active they are in coping with the epidemic situation. Therefore, improving medical students’ cognition of COVID-19 is beneficial to their mental health. Government departments and universities should make use of social platforms, social software and other new media to attract medical students to consciously receive health education on epidemic prevention measures and related knowledge in COVID-19. Similarly, compared to the low symptoms group, the risk of mental health problems in the poor mental health group with fear of being infected with COVID-19 was three times higher than that in students without this fear. These results indicated that the outbreak of COVID-19 might have a significant effect on the risk of mental health in medical students. This was consistent with previous studies conducted in Guangzhou, which suggested that psychological consequences of the COVID-19 could be serious in college students [63]. Under the stress of the COVID-19 epidemic, the mental health status of medical students had clustering characteristics. It is urgent to implement targeted psychological interventions and health education measures according to the latent group.

Nevertheless, the present study had several potential limitations. First, this was a cross-sectional study, thereby precluding conclusions on causality and weakening the dynamic analysis of mental health problems in medical students. Second, the instruments measuring the mental health used in our study were all conducted using self-rating scales, which may influence the accuracy of the results. Third, the medical students’ mental health problems included not only depression and anxiety, but also other psychological problems that were not taken into consideration in our study. This may lead to underestimation of medical students’ psychological problems.

In conclusion, this is the first study using LCA to explore mental health subgroups of medical students during the COVID-19 epidemic. LCA is a useful tool for studying and classifying mental health at the population level. It was found that the mental health status of medical students had clustering characteristics. The results will be highly relevant to medical education and could be a very important reminder of the current mental health status of medical students.

## Data Availability

The data that support the findings of this study are available from the corresponding author upon reasonable request.
